# Subjective well-being in non-obese individuals depends strongly on body composition

**DOI:** 10.1038/s41598-021-01205-6

**Published:** 2021-11-08

**Authors:** Małgorzata Chlabicz, Marlena Dubatówka, Jacek Jamiołkowski, Paweł Sowa, Magda Łapińska, Andrzej Raczkowski, Wojciech Łaguna, Anna M. Moniuszko-Malinowska, Napoleon Waszkiewicz, Irina Kowalska, Karol A. Kamiński

**Affiliations:** 1 Department of Population Medicine and Lifestyle Diseases Prevention, Medical University of Białystok, ul. Waszyngtona 13A, 15-269 Białystok, Poland; 2 Department of Invasive Cardiology, Medical University of Białystok, Białystok, Poland; 3 Faculty of Computer Science, Bialystok University of Technology, Białystok, Poland; 4 Department of Infectious Diseases and Neuroinfection, Medical University of Białystok, Białystok, Poland; 5 Department of Psychiatry, Medical University of Białystok, Białystok, Poland; 6 Department of Internal Medicine and Metabolic Diseases, Medical University of Białystok, Białystok, Poland; 7 Department of Cardiology, Medical University of Białystok, Białystok, Poland

**Keywords:** Epidemiology, Human behaviour

## Abstract

While obesity has been correlated with welfare in the general population, there is not much data on the influence of body composition on welfare among the non-obese adult individuals. In this study, a total of 726 non-obese individuals from the general population were analyzed. The mean age was 46.8 ± 15.4 years and 42.1% of participants were male. The anthropometric measurements and dual energy X-ray absorptiometry (DEXA) were done. The mean value for the Satisfaction with Life Scale (SWLS) was 23.09 ± 5.43, for Euro Quality of Life Visual Analogue Scale (EQ-VAS) was 78.0 ± 14.5, and for the Beck Depression Inventory (BDI) was 6.7 ± 6.6. On the SWLS, the higher waist-hip ratio had a negative impact even after adjusting for age, gender, and concomitant diseases. EQ-VAS was inversely associated with android fat distribution and directly associated with muscle mass. BDI value was inversely associated with lower muscle mass, especially in lower limbs. The well-being of women was mainly associated with the distribution of adipose tissue and less with the distribution of muscle tissue—abdominal fat distribution has a particularly negative impact on well-being among women. In contrast, men’s well-being depends more on muscle mass and to a lesser extent on the distribution of fat tissue—a positive significant effect has lean mass and a circumference of thigh below gluteal fold.

## Introduction

Life satisfaction (LS) is the goal of human development and is very important to subjective well-being and psychosocial functioning^1^, and due to LS, well-being assessment is an important scientific task.

The Satisfaction With Life Scale (SWLS) was developed in 1959 by Diener^2^ to measure the cognitive aspect of subjective well-being. The SWLS has a recognized validity related to but still separate from constructs such as depression, negative and positive effects, self-esteem, anxiety, and psychological distress. The scores on the SWLS have been shown to correlate with measures of mental health and be predictive of future behaviors.

The Euro Quality of Life Visual Analogue Scale (EQ-5D) was designed to measure health related quality of life (HRQL)^3^. This instrument is widely used in the health sector: in patient-reported outcome exercises, in population heath studies, and in health technology assessment^3^. The Visual Analogue Scale (EQ-VAS) is the second part of our questionnaire, asking to mark health status on the day of the interview on a 20 cm vertical scale with end points of 0 and 100.

Additionally, depression is the most common psychiatric disorder, which is related to life satisfaction. The most common scale used to asses this disorder is the Beck Depression Inventory^4^ (BDI). The BDI is a 21-item, multi-choice self-report rating inventory that measures characteristic attitudes and symptoms of depression.

Several previous studies have shown obesity to be related to the deterioration in LS^5,6^, HRQL^7–9^ and severity of depression^10–12^. While obesity has been correlated with welfare in the general population, there is not much data on the influence of body composition on welfare among the non-obese adult individuals. Therefore, body composition is presumed to play an important role in the genesis of mental disorders. Biological pathways are known to relate body composition and brain function. One of the hypotheses that explains the effect of body composition on LS is the hypothalamic–pituitary–adrenal (HPA) axis dysregulation. The hypercortisolism, directing storage fat to central adipose tissue depots, and is inversely related to psychological quality of life. The second hypothesis relates to brain-derived neurotrophic factor (BDNF). BDNF drives neurogenesis in the hippocampus and is produced in skeletal muscle. A decreased contraction of skeletal muscle can cause a decline in secretion of BDNF as well as a volume reduction of the hippocampus and thus, has been implicated in LS. Third hypothesis is sociological effects. The ideal body image based on media and social media is very important nowadays and can affect the quality of life regardless of the metabolic background. Some studies have shown that the percentage of fat was inversely associated with satisfaction with body image more strongly than the body mass index (BMI)^13^. We hypothesized that the body composition, i.e. muscle mass, adipose tissue mass, android fat distribution, leg muscle mass and thigh circumference could be related with LS, HRQL and severity of depression syndrome.

### Aim of the study

We aimed to investigate the relationship between body composition and subjective well-being in non-obese adult individuals from the general population using the Satisfaction with Life Scale (SWLS), the Euro Quality of Life Visual Analogue Scale (EQ-VAS) and the Beck Depression Inventory (BDI).

## Patients and methods

### Study population

The study was conducted in 2017–2020 in a representative sample of area residents aged 20–79. Overall, 2449 randomly selected residents from the mayor’s office database were invited to participate in the study and 966 individuals were examined. Due to obesity (BMI ≥ 30 kg/m^2^), 240 individuals were excluded from further analysis. As a result, 726 people (mean age 46.76 ± 15.36 years, 42.1% male) were included in the research group.

### Data collection and assays

The data was collected through standardized health examinations in a specially equipped examination center. The details of the subjects’ medical history were collected from questionnaires. The anthropometric measurements were taken, including height, weight, circumferences of waist, hips, thigh (just below the buttock fold) using the SECA 201 tape (SECA, Hamburg, Germany) with participants wearing light clothing without shoes. Measurements were performed in accordance with the World Health Organization (WHO) guidelines^14^. The waist-to-hip ratio (WHR) was calculated as the ratio of the circumference between the waist and hips. Body mass index (BMI) was calculated as weight in kilograms divided by height in meters squared.

The body composition was measured by dual energy X-ray absorptiometry (DEXA) (GE Healthcare, Chicago, Illinois, USA). The gynoid (G) fat (GF) and lean (GL), android (A) fat (AF) and lean (AL), legs (L) fat (LF) and lean (LL) were measured automatically as described previously^15^. Fat mass index (FMI), lean mass index (LMI) and Visceral Mass Index (VMI) were calculated as fat, lean and visceral mass in kilograms divided by height in meters squared. The AF/GF ratio was calculated between the fat of the android and fat of the gynoid regions. The GF/total fat (GF/TF) ratio was calculated as ratio between the gynoid fat and total fat. The AF/total fat (AF/TF) ratio was calculated as ratio between the android fat and total fat. The leg fat/total fat (LF/TF) ratio was calculated as ratio between the leg fat and total fat. The ratio of lean GL/total lean (GL/TL), AL/total lean (AL/TL) and legs lean/total lean (LL/TL) were calculated analogously.

The Satisfaction with Life Scale (SWLS)^16^ has been used to measure a life satisfaction component of subjective well-being. The scale is a 5-item questionnaire rated on a 7-point scale from 1—strongly disagree to 7—strongly agree. The possible range of scores is 5–35, with a score of 20 representing a neutral point on the scale. Scores between 5 and 9 indicate that a respondent is extremely dissatisfied with life, whereas scores between 31 and 35 indicate that a respondent is extremely satisfied.

Euro Quality of Life Visual Analogue Scale (EQ-5D) was designed to measure health related quality of life^3^. Visual Analogue Scale (EQ-VAS) is the second part of the questionnaire, asking patients to mark health status on the day of the interview on a 20 cm vertical scale with end points of 0 and 100. The bottom rate (0) corresponds to “the worst health you can imagine”, and the highest rate (100) corresponds to “the best health you can imagine”. This information can be used as a quantitative measure of health outcome as judged by individual respondents.

Depression symptoms were assessed by the Beck Depression Inventory^4^ (BDI), a self-report measurement to assess severity of depression. The scale comprises of 21 questions with four answer options for each question. Each answer recorded scored on a scale of 0 to 3. Higher total scores indicate more severe depressive symptoms.

### Ethical issues

Ethical approval for this study was provided by the Ethics Committee of the Medical University of Bialystok (Poland) on 31 March 2016 (approval number: R-I-002/108/2016). The study was conducted in accordance with the Declaration of Helsinki and all participants gave written informed consent.

### Statistical analysis

Descriptive statistics for quantitative variables were presented as means and standard deviations and as counts and frequencies for qualitative variables. Comparisons of continuous variables between subgroups were conducted using the Mann–Whitney or Fisher’s tests. Associations between SWLS, EQ-VAS, BDI scales and body composition variables were analyzed by gender using simple and multiple linear regression models. Multiple linear regression models included single variables listed in the tables and were adjusted for age (Model 1), history of CVD (i.e. arterial hypertension (AH), atrial fibrillation (AF), MI, coronary heart disease (CHD), peripheral artery disease (PAD), stroke) and history of diabetes mellitus (DM) (Model 2) or for age and WHR (Model 3). The analysis was performed in gender disaggregated populations. The charts present standardized plots of the regression equation with 95% confidence intervals. Statistical hypotheses were verified at 0.05 significance level. The IBM SPSS Statistics 20.0 statistical software (Armonk, NY, USA) was used for all calculations.

## Results

The baseline characteristics and the characteristics of participants according to sex are summarized in Table 1.

**Table 1:** Characteristics of the non-obese general population.

Variable	Total population *n* = 726	Women *n* = 420	Men *n* = 306	p-values*
Age, years	46.76 ± 15.36	47.48 ± 15.29	45.78 ± 15.43	0.11
SWLS	23.09 ± 5.43	22.79 ± 5.57	23.49 ± 5.22	0.06
EQ-VAS	77.95 ± 14.49	76.42 ± 14.83	80.03 ± 13.80	< 0.001
BDI	6.66 ± 6.55	7.45 ± 6.69	5.58 ± 6.21	< 0.001
Height, cm	170.23 ± 9.56	164.48 ± 6.36	178.12 ± 7.33	< 0.001
Weight, kg	71.64 ± 12.69	64.65 ± 8.74	81.23 ± 10.88	< 0.001
Waist, cm	81.97 ± 10.63	76.64 ± 8.38	89.32 ± 8.89	< 0.001
Hip, cm	95.99 ± 7.11	95.83 ± 7.63	96.20 ± 6.32	0.47
Thigh, cm	56.40 ± 4.79	56.19 ± 4.92	56.69 ± 4.59	0.11
BMI, kg/m^2^	24.62 ± 3.11	23.93 ± 3.18	25.56 ± 2.76	< 0.001
BMI < 25 kg/m^2^	377 (51.92)	260 (62)	117 (38)	< 0.001
BMI 25–29.99 kg/m^2^	349 (48.07)	160 (38)	189 (62)	< 0.001
WHR	0.85 ± 0.09	0.80 ± 0.07	0.93 ± 0.07	< 0.001
WHR, ≥ 0.85 women, ≥ 0.9 men	299 (41.18)	94 (22)	205 (67)	< 0.001
Lean Mass Index, (kg/m^2^)	16.10 ± 2.02	14.80 ± 1.25	17.86 ± 1.45	< 0.001
Fat Mass Index, (kg/m^2^)	7.77 ± 2.37	8.40 ± 2.34	6.91 ± 2.13	< 0.001
Android fat mass, kg	1.91 ± 0.88	1.72 ± 0.75	2.17 ± 0.97	< 0.001
Gynoid fat mass, kg	3.60 ± 1.05	3.98 ± 0.98	3.08 ± 0.91	< 0.001
Gynoid lean mass, kg	7.0 ± 1.51	5.96 ± 0.69	8.43 ± 1.11	< 0.001
Legs fat mass, kg	6.89 ± 2.18	7.85 ± 2.00	5.60 ± 1.67	< 0.001
Legs lean mass, kg	16.16 ± 3.66	13.71 ± 1.85	19.51 ± 2.76	< 0.001
AF/GF	0.54 ± 0.22	0.42 ± 0.15	0.69 ± 0.21	< 0.001
GF/TF	0.16 ± 0.07	0.16 ± 0.07	0.15 ± 0.08	< 0.001
AF/TF	0.08 ± 0.06	0.07 ± 0.04	0.10 ± 0.07	< 0.001
LF/TF	0.30 ± 0.14	0.32 ± 0.14	0.27 ± 0.14	< 0.001
GL/TL	0.15 ± 0.05	0.13 ± 0.03	0.18 ± 0.05	< 0.001
LL/TL	0.34 ± 0.11	0.29 ± 0.08	0.42 ± 0.11	< 0.001

The data is shown as n (%), mean ± SD.

*SD* standard deviation, *SWLS* Satisfaction with Life Scale, *EQ-VAS* Euro Quality of Life Visual Analogue Scale, *BDI* Beck Depression Inventory, *BMI* Body Mass Index, *kg* kilogram, *m*^*2*^ square meter, *WHR* waist-hip ratio, *AF/GF* android fat/gynoid fat, *GF/TF* gynoid fat/total fat, *AF/TF* android fat/total fat, *LF/TF* legs fat/total fat, *GL/TL* gynoid lean/total lean, *LL/TL* legs lean/total lean.

*p-values for comparing men and women.

### Female population

In the female population sample, a significant negative association between the SWLS and android fat distribution parameters was observed while height and gynoid lean mass (GL) were positively associated with the SWLS. After adjustment for age (Model 1), only the relation between the SWLS and WHR remained significant. Finally, higher WHR had a negative effect on the SWLS even after adjustment for age and comorbidities (Model 2). After adjustment for age and WHR (Model 3), higher GF/TF ratio presented a negative impact on the SWLS.

EQ-VAS showed a significant negative association with higher age, weight, BMI, FMI, LMI and android fat distribution parameters while height was positively associated with EQ-VAS. The negative association between EQ-VAS and BMI, FMI, abdominal fat distribution remained significant after adjustment for age (Model 1). Finally, FMI and android fat distribution parameters were inversely associated with even EQ-VAS after adjustment for age and history of CVD and DM (Model 2). In Model 3, FMI and android fat distribution parameters (VMI, AF) remained significantly associated after adjustment for age and WHR.

With BDI scoring, age and android fat distribution parameters were positively associated, while height and lean mass parameters presented a significant negative association. In Model 1, the positive relationship between the BDI and WHR and inverse relationship with GL/TL, LL/TL remained significant. However, in Model 2 and Model 3, only GL/TL and LL/TL remained significant.

**Figure 1:**
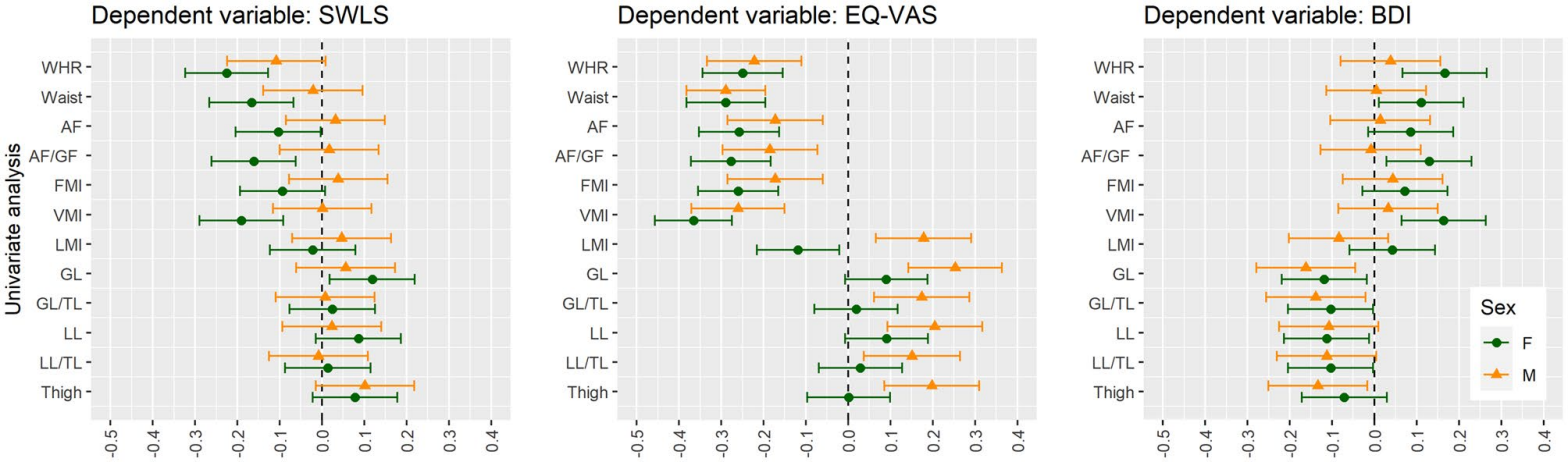
Univariate linear regression analysis between body composition and subjective well-being scores in non-obese women and men. *SWLS* Satisfaction with Life Scale, *EQ-VAS* Euro Quality of Life Visual Analogue Scale, *BDI* Beck Depression Inventory, *WHR* waist-hip ratio, *AF* android fat, *AF/GF* android fat/gynoid fat, *FMI* Fat Mass Index, *VMI* Visceral Mass Index, *LMI* Lean Mass Index, *GL* gynoid lean, *GL/TL* gynoid lean/total lean, *LL* legs lean, *LL/TL* legs lean/total lean, *F* female, *M* male.

**Figure 2:**
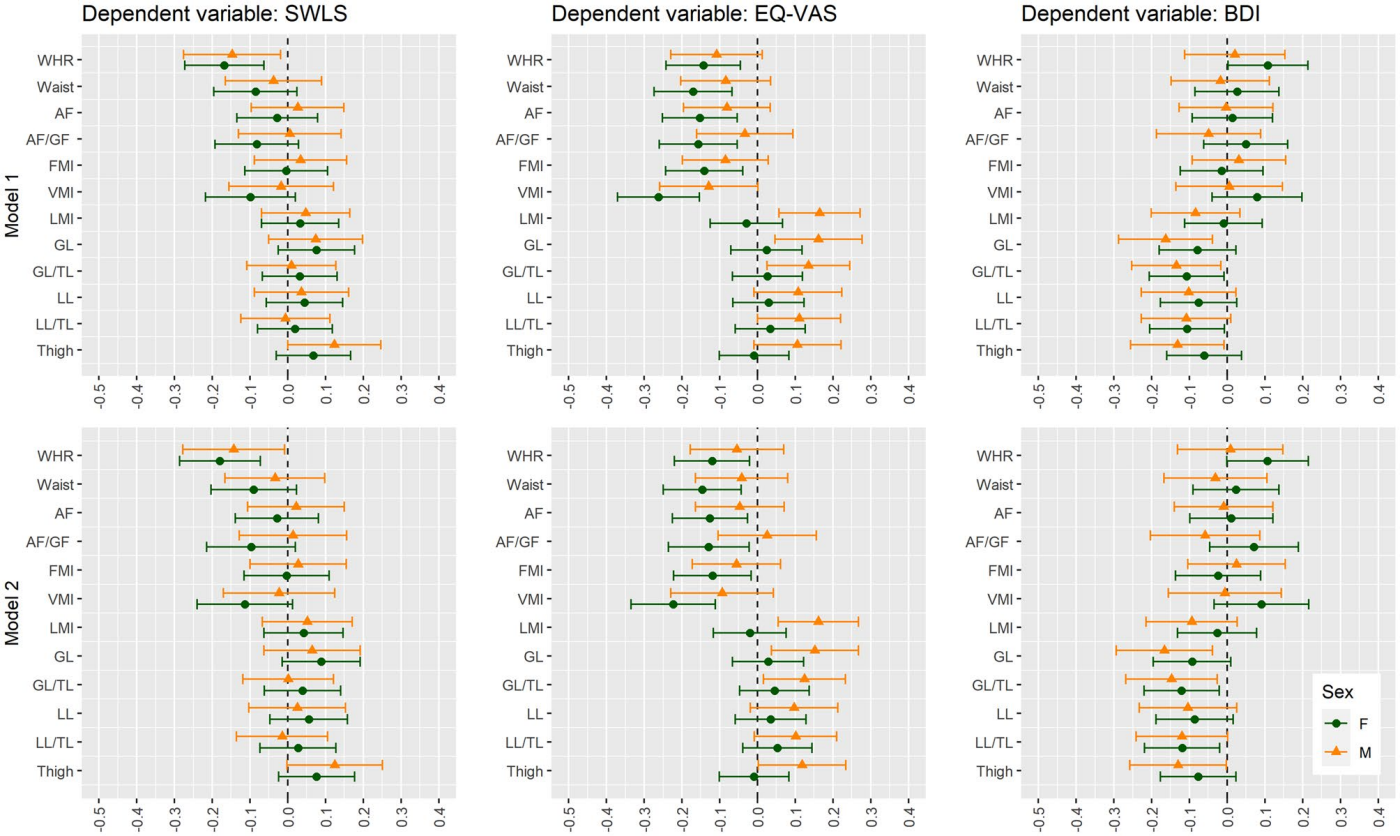
Multivariable linear regression analysis adjusted for age (Model 1) and by age, history of CVD, history of diabetes mellitus (Model 2) between body composition and subjective well-being scores in non-obese women and men. *SWLS* Satisfaction with Life Scale, *EQ-VAS* Euro Quality of Life Visual Analogue Scale, *BDI* Beck Depression Inventory, *WHR* waist-hip ratio, *AF* android fat, *AF/GF* android fat/gynoid fat, *FMI* Fat Mass Index, *VMI* Visceral Mass Index, *LMI* Lean Mass Index, *GL* gynoid lean, *GL/TL* gynoid lean/total lean, *LL* legs lean, *LL/TL* legs lean/total lean, *F* female, *M* male. Model 1: adjusted for age. Model 2: adjusted for age, history of cardiovascular diseases (i.e. arterial hypertension, atrial fibrillation, myocardial infarction, coronary heart disease, peripheral artery disease, stroke) and history of diabetes mellitus.

**Figure 3:**
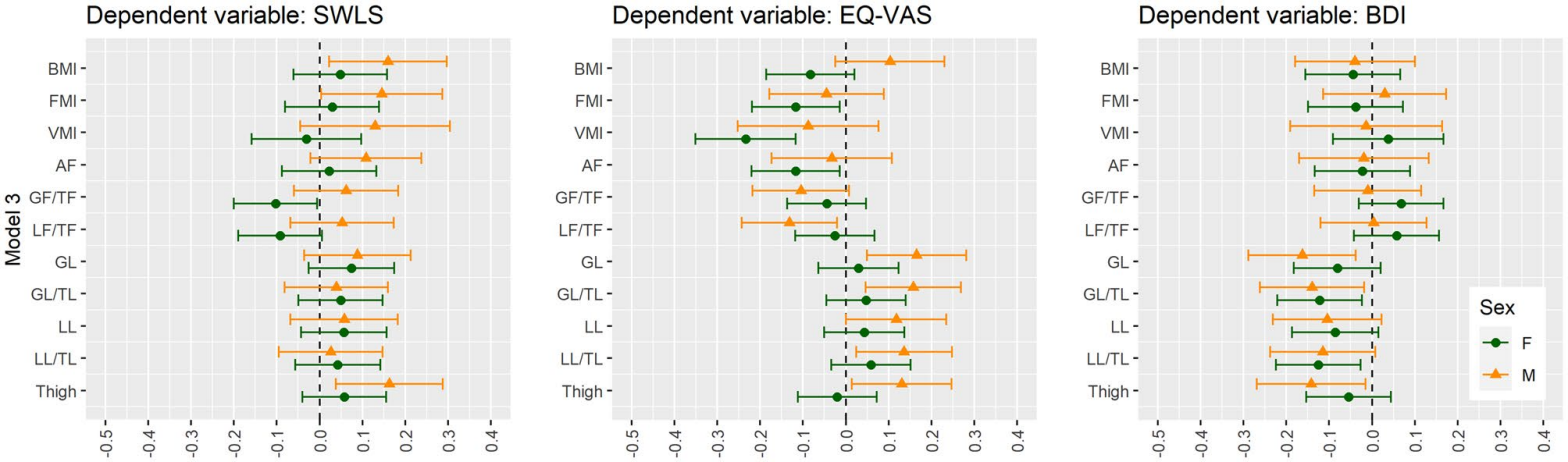
Multivariable linear regression analysis adjusted for age and WHR (Model 3) between body composition and subjective well-being scores in non-obese women and men. *SWLS* Satisfaction with Life Scale, *EQ-VAS* Euro Quality of Life Visual Analogue Scale, *BDI* Beck Depression Inventory, *BMI* Body Mass Index, *FMI* Fat Mass Index, *VMI* Visceral Mass Index, *AF* android fat, *GF/TF* gynoid fat/total fat, *LF/TF* legs fat/total fat, *GL* gynoid lean, *GL/TL* gynoid lean/total lean, *LL* legs lean, *LL/TL* legs lean/total lean, *F* female, *M* male. Model 3: adjusted for age and WHR.

The details are presented in Tables S1, S3, S5 and S7. The most significant variables are presented in Figs. 1, 2 and 3.

### Male population

In the male population sample, no association was found between the SWLS and investigated variables in univariate analysis. After adjustment for age (Model 1) and age with comorbidities (Model 2), higher WHR value was inversely associated with the SWLS while after adjustment for age and WHR (Model 3), a positive association was found with a larger silhouette (BMI, FMI, AF) and thigh circumference.

In contrast, with EQ-VAS, several parameters of anthropometric measurement and body composition measured by DEXA were significantly related. A negative relationship was found with android fat distribution parameters while a positive relationship was found with the parameters of muscle tissue (LMI), especially of the legs. In Model 1, the inverse association between EQ-VAS and android fat distribution parameters (AF/TF) remained significant after adjustment for age. Also, a positive association between EQ-VAS and LMI, GL, GL/TL remained significant. Moreover, GF/TF, LF/TF appeared to be negatively correlated with EQ-VAS. Finally, thigh circumference, parameters of muscle mass (LMI) and leg muscle mass were positively associated; and leg fat negatively associated with EQ-VAS even after adjustment for age and history of CVD and DM (Model 2). In Model 3, the parameters of muscle mass (LMI), especially of legs became more important.

A significant negative association between BDI value, muscle mass and thigh circumference has been found. In Model 1, Model 2 and Model 3, thigh circumference and AL, GL, GL/TL remained inversely associated with BDI value.

The details are presented in Tables S2, S4, S6 and S8. The most significant variables are presented in Figs. 1, 2 and 3.

To disentangle the independent relationships of body composition in non-obese individuals with subjective well-being from any additional confounding diseases, another sub analysis was performed (Supplementary Materials). From the above analyzed population, we excluded people with a history of CVD (AH, MI, CHD, PAD and stroke), AF, heart failure (HF), DM, chronic obstructive pulmonary disease (COPD), asthma, cancer, chronic kidney disease (CKD) with glomerular filtration rate (GFR) < 60 ml/min, and any mental disorders. The baseline characteristics of this subpopulation are summarized in Table S9. In the univariate analysis in the group of women in which very similar parameters were related to the scales, no new statistically significant parameters appeared (Table S10). In Model 1, more parameters of android fat distribution were associated with subjective well-being. Namely, the SWLS scores were negatively associated with VTI (B − 0.833, p = 0.005), AF/GF (B − 9.323, p = 0.003) and AF/TF (B − 25.418, p = 0.010). However, in men, the relationship between well-being and higher muscle mass was even more pronounced—there were new significant positive relationships with the SWLS: the thigh circumference, the hip circumference and gynoid lean mass (Table S11). After taking into account age and gender, WHR (B − 15.819; p = 0.001) remained negatively significant in relation to the SWLS, with concomitant positive relation with thigh circumference (B 0.236, p = 0.009). Similarly, in the EQ-VAS analysis in men, an additional strong positive link appeared with thigh circumference in Model 1. In depression analysis in men, a stronger negative link with low muscle mass, especially in lower limbs, was underlined. In conclusion, in this sub-population, comparable factors in women were associated with subjective well-being, while in men, a more positive relationship with thigh circumference was emphasized. Detailed analyses can be found in the Supplementary Materials in Tables S12–S13.

## Discussion

The present study reports that body composition has an impact on well-being in non-obese individuals from the general population sample. We observed that different dimensions are related to different well-being data measures and gender. The higher WHR value had the greatest negative impact on life satisfaction (SWLS) even after adjustment for age, gender, and concomitant diseases. Health related quality of life (EQ-VAS) was inversely associated with android fat distribution and directly associated with muscle mass, especially in lower limbs. Depressive symptoms (assessed by the BDI) were associated with lower muscle mass, especially in lower limbs. The well-being of women associated mainly with the distribution of adipose tissue and less with the distribution of muscle tissue—abdominal fat distribution has a particularly negative impact on well-being among women. In contrast, men’s well-being depends more on muscle mass and, to a lesser extent, on the distribution of fat tissue.

### Life satisfaction

LS is a crucial indicator of people’s quality of life and reflects a subjective judgment on the quality of life based on individual criteria for happiness and success. In men, none of the analyzed parameters influenced LS in the univariate analysis. Only after correction for age, a negative impact of WHR on LS. Rosmond et al.^17^ in univariate analyses showed that BMI and WHR correlated with symptoms of depression and anxiety, sleep disturbances, psychosomatic diseases as well as the degree of life satisfaction. When adjusted for WHR, all significant relationships with BMI disappeared. In contrast, the WHR adjusted for BMI showed remaining significant associations with the use of anxiolytics, hypnotics, antidepressant drugs, degree of melancholy and life satisfaction (p = 0.002, negative) and dyspepsia (p < 0.001). It was concluded that in contrast to BMI, the WHR is associated with symptoms of depression and anxiety with associated sleep disturbances as well as psychosomatic symptoms and dissatisfaction. The researchers obtained similar results in both groups of men and women^18^. It was hypothesized that the mechanism involved might be an increased secretion of cortisol, directing storage fat to central adipose tissue depots. A study by Tang et al.^19^ confirmed that cortisol levels were inversely related to psychological quality of life. The results indicate that activity of the hypothalamic–pituitary–adrenal (HPA) axis is linked to LS. In our study, the WHR had the greatest negative association with LS, which may suggest such a relationship also in the non-obese population, regardless of gender.

Moreover, in multivariate analysis adjusted for age and WHR, we demonstrated a positive association of a larger silhouette (BMI, FMI, android fat mass and thigh circumference) with LS in men. Calzo et al.^20^ showed, that the desire for bigger muscles increased slightly each year across adolescent males (β = 0.10, 95% C.I. 0.09, 0.11), and attempts to gain weight increased three-fold across adolescence with up to 30% reporting weight gain attempts by age 16. While media images and advertisements reinforce the notion that thinness is central to women’s physical attractiveness, content analyses have demonstrated that boys and men are inundated with images of unattainable muscularity^21,22^.

In women, the multiple parameters of the abdominal fat distribution influenced LS, and WHR remained the most important factors after adjusting for age, history of CVD and DM. Simultaneously, in multivariate analysis adjusted for age and WHR, we demonstrated a negative effect of gynoid fat (G/TF). As mentioned above, the media often reinforce the message that slenderness is a symbol of attractiveness and beauty. This could serve as a reason for higher body dissatisfaction even in non-obese women with gynoid fat distribution.

To the best of our knowledge, this is the first analysis to show a relationship between WHR and LS in both sexes in non-obese individuals from the general population sample followed by a positive association with a larger silhouette in men, and a negative association with gynoid adipose distribution in women using WHR as a covariate.

### HRQL

Several studies showed a negative impact of obesity on HRQL^7,23,24^. In current non-obese population sample, the EQ-VAS value was inversely associated with android fat distribution and directly associated with muscle mass, especially in lower limbs. The relationship between android fat distribution and HRQL would be explained similarly to LS. Increasingly, HRQL is considered to be an important outcome of care for HPA axis dysregulation. Bucy et al.^25^ showed that hypercortisolism had the greatest negative influence on HRQL with pituitary and benign adrenal causes of Cushing’s syndrome and primary adrenal insufficiency patients. The relationship between muscle mass and HRQL has also been shown by other researchers, Kim et al.^26^ showed that involuntary weight loss combined with low muscle mass was more closely associated with poor HRQL than involuntary weight loss alone in older adults. Balogun et al.^27^ described the associations of low muscle mass, handgrip, and lower-limb muscle strength with HRQL in older adults. Participants with lower limb muscle strength had clinically meaningful reductions in HRQL compared to those with normal strength. We confirmed the strong positive relationship between muscle mass and HRQL, especially in lower limbs, in both sexes, but more pronounced in men. However, HRQL was negatively influenced to a greater extent by the abdominal distribution of adipose tissue in women.

### Depression

Several studies showed a correlation between obesity and depression^10–12,28^. Koksal et al.^12^ showed that body fat percentage had the highest correlation with depression severity in obese patients; however, a positive association was found with BMI, waist circumference, hip circumference, visceral fat percentage^12^. Speed et al.^11^, showed that fat mass and short stature are causal risk factors for depression. A study in overweight and obese individuals with metabolic syndrome showed that a higher percentage of body fat and lower total lean mass were associated with an increased severity of depression and anxiety^29^. The authors hypothesized that an increase in lean mass may indicate healthier individuals. In our study, short stature was a risk factor for depression only in women using univariate regression analysis. We did not show a relationship between the severity of depression and BMI or FMI; however, we demonstrated a relationship with an android fat distribution in women and low muscle mass in both sexes regardless of the comorbidities. This observation is consistent with previous work by Moon et al.^30^ who reported that girls with decreased muscle mass had a greater tendency for a depressed mood compared to girls with optimal muscle mass (p = 0.023). Likewise, Heo et al.^31^ showed the independent association between low skeletal muscle mass and depressive symptoms in middle-aged men. Noh and Park^32^ examined participants aged 65 years or older (*n* = 3219), and showed that for men, handgrip strength and relative handgrip strength were inversely associated with risk of stress, depressed mood, and suicidal ideation. Biological pathways are known to affect a decline in muscle mass and brain function. Brain-derived neurotrophic factor (BDNF) drives neurogenesis in the hippocampus and is produced in skeletal muscle^33^. A decreased contraction of skeletal muscle can cause a decline in secretion of BDNF as well as a volume reduction of the hippocampus and thus, has been implicated in psychiatric illness^34^. Furthermore, inflammation and oxidative stress are common pathophysiology of reduced muscle mass and cause depression. The activity of skeletal muscle boosts the immune system, and its redox affects reduced muscle catabolism and maintains mood^35^. However, we showed that WHR was significantly associated with depression in the female population sample. It seems that different aspects of depression have abdominal fat distribution in female population. Alshehri et al.^36^ showed that overall and abdominal adiposity measures were associated with a depressive mood. Rivenes et al.^37^ showed that elevated WHR was associated with an increased prevalence of depression. After adjustment for BMI, physical activity, social isolation, and somatic diseases, WHR remained independently associated with depression in both males and females. A clinical implication of this finding was that abdominal fat distribution appears to be the key mediator in the relationship between obesity and depression, and increased BMI was not independently associated with depression. The authors conclude that these findings were consistent with a hypothesis that links obesity and depression via metabolic disturbances involving the HPA axis. In the current study, we were able to detect a negative association between lower lean mass and a related circumference of thigh and depressive mood in men in contrast to android fat distribution and lower muscle mass of legs in women. These results may imply that various factors of body composition play a crucial role in relation to depression in a non-obese population categorized by gender.

### Population

Overall, 240 individuals with obesity (BMI ≥ 30 kg/m^2^) were excluded from our research. Since being overweight did not significantly affect HRQL scores^38^, we did not exclude overweight participants from the study. To disentangle the independent relationships of body composition in non-obese individuals with subjective well-being from any additional confounding diseases, another sub analysis was performed, confirming our prior analysis. Consequently, no other diseases negatively affected well-being but body composition was significantly associated with welfare.

### Limitations

There are some limitations associated with the present study. Firstly, this study is limited to a sample from an urban environment, and a relatively low (41.7%) participation rate could have affected the representativeness of the study. Secondly, to analyze the quality of life, only visual analogue scale presenting the second part of the Euro Quality of Life Visual Analogue Scale (EQ-5D) (EQ-VAS) was used.

## Conclusions

Our main findings point out that the body composition has an impact on well-being in non-obese individuals from general population. These associations differ depending on particular aspects of self-reported well-being and gender. Abdominal obesity measured by WHR has the greatest negative impact on life satisfaction even after adjustment for age, gender and concomitant diseases. Health related quality of life is inversely associated with android fat distribution and directly associated with muscle mass. BDI value is associated with low muscle mass, especially in lower limbs. The well-being of women is associated mainly with the distribution of adipose tissue and less with the distribution of muscle tissue—abdominal fat distribution has a particularly negative impact. In contrast, men’s well-being depends more on muscle mass and, to a lesser extent, on the distribution of fat tissue. These results suggest that HPA-axis dysregulation most likely has a greater impact in the female population, and brain-derived neurotrophic factor (BDNF) may have a greater association in the male population. Whereas, the sociological impact on LS seems to be of secondary importance in both sexes.

## Supplementary Information

Supplementary Information.
