# Supplementary material for: Subjective well-being in non-obese individuals depends strongly on body composition
Source: Sci Rep. 2021 Nov 8;11:21797. doi: 10.1038/s41598-021-01205-6 (PMC8576020; doi:10.1038/s41598-021-01205-6)
Supplement: Supplementary file 1 — Supplementary Information. [file 41598_2021_1205_MOESM1_ESM.pdf]

Subjective well-being in non-obese individuals depends strongly on body composition

Małgorzata Chlabicz[1,2], Marlena Dubatówka[1], Jacek Jamiołkowski[1], Paweł Sowa[1] , Magda Łapińska[1], Andrzej Raczkowski[1], Wojciech Łaguna[3], Anna M. Moniuszko-Malinowska[4], Napoleon Waszkiewicz[5], Irina Kowalska[6] and Karol A. Kamiński[1,7]*

[1]Department of Population Medicine and Lifestyle Diseases Prevention, Medical University of Białystok, Białystok, Poland

[2] Department of Invasive Cardiology, Medical University of Białystok, Białystok, Poland

[3] Faculty of Computer Science, Bialystok University of Technology, Białystok, Poland

[4]Department of Infectious Diseases and Neuroinfection, Medical University of Białystok, Białystok, Poland

[5]Department of Psychiatry, Medical University of Białystok, Białystok, Poland

[6]Department of Internal Medicine and Metabolic Diseases, Medical University of Białystok, Białystok, Poland

[7]Department of Cardiology, Medical University of Białystok, Białystok, Poland

*Corresponding author:

Karol Adam Kamiński, MD, PhD

Department of Population Medicine and Lifestyle Diseases Prevention, Medical University of Białystok

ul. Waszyngtona 13A, 15-269 Białystok, Poland

Tel.: +48-856-865-371

fizklin@wp.pl

Table S1. Univariate linear regression analysis between body composition and subjective well-being scores in non-obese women.

Variable	SWLS		EQ-VAS		BDI	
	B	*p*	B	*p*	B	*p*
Age, years	-0.079	<0.001	-0.327	<0.001	0.086	<0.001
Height, cm	0.091	0.040	0.455	<0.001	-0.152	0.004
Weight, kg	-0.010	0.770	-0.190	0.023	-0.014	0.725
BMI, kg/m^2	-0.143	0.109	-1.102	<0.001	0.145	0.178
WHR	-18.226	<0.001	-54.015	<0.001	16.017	0.001
Waist, cm	-0.110	0.001	-0.514	<0.001	0.088	0.030
Hip, cm	0.009	0.801	-0.233	<0.001	-0.023	0.626
Thigh, cm	0.088	0.129	0.004	0.981	-0.097	0.164
Fat Mass Index, kg/m^2	-0.220	0.069	-1.660	<0.001	0.204	0.161
Lean Mass Index, kg/m^2	-0.098	0.668	-1.403	0.017	0.224	0.417
Visceral mass index, kg/m^2	-6.139	<0.001	-31.455	<0.001	6.235	0.001
Android fat mass, kg	-0.001	0.044	-0.005	<0.001	0.001	0.097
Gynoid fat mass, kg	8.553	0.770	-0.001	0.054	0.000	0.572
Gynoid lean mass, kg	0.001	0.021	0.002	0.072	-0.001	0.021
Legs fat mass, kg	0.000	0.265	0.000	0.408	0.000	0.183
Legs lean mass, kg	0.000	0.095	0.001	0.070	0.000<br>β<br>-0.113	0.028
AF/GF	-6.196	0.002	-28.203	<0.001	5.867	0.012
GF/TF	-7.221	0.074	-5.097	0.619	5.428	0.278
AF/TF	-20.562	0.005	-65.676	<0.001	19.841	0.024
LF/TF	-2.805	0.163	0.239	0.963	1.839	0.464
GL/TL	4.373	0.637	8.927	0.710	-22.374	0.045
LL/TL	1.024	0.787	5.687	0.564	-9.202	0.044

AF/GF, android fat/gynoid fat; GF/TF, gynoid fat/total fat; AF/TF, android fat/total fat; LF/TF legs fat/total fat; GL/TL gynoid lean/total lean; LL/TL legs lean/total lean

Table S2. Univariate linear regression analysis between body composition and subjective well-being scores in non-obese men.

Variable	SWLS		EQ-VAS		BDI	
	B	*p*	B	*p*	B	*p*
Age, years	0.008	0.677	-0.275	<0.001	0.021	0.387
Height, cm	0.001	0.988	0.297	0.007	-0.090	0.082
Weight, kg	0.023	0.420	0.087	0.246	-0.040	0.235
BMI, kg/m^2	0.097	0.389	-0.196	0.507	-0.021	0.879
WHR	-8.377	0.069	-45.278	<0.001	3.488	0.532
Waist, cm	-0.013	0.718	-0.303	<0.001	0.003	0.941
Hip, cm	0.067	0.168	-0.098	0.441	-0.032	0.579
Thigh, cm	0.115	0.089	0.596	0.001	-0.181	0.026
Fat Mass Index, kg/m^2	0.092	0.521	-1.114	0.003	0.125	0.470
Lean Mass Index, kg/m^2	0.167	0.439	1.711	0.002	-0.366	0.160
Visceral mass index, kg/m^2	0.016	0.989	-13.570	<0.001	0.750	0.593
Android fat mass, kg	0.000	0.599	-0.002	0.003	8.499	0.823
Gynoid fat mass, kg	0.000	0.291	-0.001	0.481	3.748	0.925
Gynoid lean mass, kg	0.000	0.348	0.003	<0.001	-0.001	0.007
Legs fat mass, kg	0.000	0.323	0.000	0.365	6.120	0.779
Legs lean mass, kg	4.344	0.699	0.001	<0.001	0.000	0.073
AF/GF	0.418	0.776	-11.995	0.001	-0.261	0.883
GF/TF	1.146	0.777	-16.183	0.123	-0.656	0.892
AF/TF	1.414	0.757	-34.338	0.004	-1.144	0.834
LF/TF	0.584	0.792	-10.235	0.074	0.092	0.972
GL/TL	0.816	0.903	50.396	0.003	-18.386	0.021
LL/TL	-0.402	0.886	18.514	0.010	-6.312	0.061

AF/GF, android fat/gynoid fat; GF/TF, gynoid fat/total fat; AF/TF, android fat/total fat; LF/TF legs fat/total fat; GL/TL gynoid lean/total lean; AL/TL android lean/total lean; LL/TL legs lean/total lean

Table S3. Multivariable linear regression analysis adjusted by age (Model 1) between body composition and subjective well-being scores in non-obese women.

Variable	SWLS		EQ-VAS		BDI	
	B	*p*	B	*p*	B	*p*
Height, cm	0.008	0.873	0.131	0.287	-0.073	0.219
Weight, kg	0.009	0.775	-0.106	0.187	-0.034	0.373
BMI, kg/m^2	0.012	0.899	-0.540	0.025	-0.030	0.799
WHR	-13.594	0.002	-31.031	0.005	10.400	0.046
Waist, cm	-0.057	0.129	-0.303	0.001	0.021	0.643
Hip, cm	0.046	0.213	-0.093	0.323	-0.063	0.158
Thigh, cm	0.076	0.178	-0.028	0.844	-0.083	0.226
Fat Mass Index, kg/m^2	-0.010	0.939	-0.901	0.007	-0.041	0.798
Lean Mass Index, kg/m^2	0.146	0.526	-0.351	0.546	-0.052	0.853
Visceral mass index, kg/m^2	-3.180	0.105	-22.548	<0.001	3.013	0.194
Android fat mass, kg	0.000	0.608	-0.003	0.003	0.000	0.801
Gynoid fat mass, kg	0.000	0.530	-0.001	0.141	0.000	0.400
Gynoid lean mass, kg	0.001	0.143	0.001	0.618	-0.001	0.129
Legs fat mass, kg	0.000	0.181	0.000	0.654	0.000	0.130
Legs lean mass, kg	0.000	0.395	0.000	0.545	0.000	0.143
AF/GF	-3.145	0.147	-15.929	0.003	2.237	0.387
GF/TF	-7.482	0.058	-7.399	0.444	6.059	0.218
AF/TF	-13.468	0.068	-35.560	0.052	11.678	0.194
LF/TF	-3.054	0.121	-1.197	0.805	2.290	0.353
GL/TL	5.655	0.533	12.487	0.581	-23.279	0.034
LL/TL	1.383	0.710	6.600	0.478	-9.438	0.035

AF/GF, android fat/gynoid fat; GF/TF, gynoid fat/total fat; AF/TF, android fat/total fat; LF/TF legs fat/total fat; GL/TL gynoid lean/total lean; AL/TL android lean/total lean; LL/TL legs lean/total lean

Model 1: adjusted for age.

Table S4. Multivariable linear regression analysis adjusted by age (Model 1) between body composition and subjective well-being scores in non-obese men.

Variable	SWLS		EQ-5D		BDI	
	B	*p*	B	*p*	B	*p*
Height, cm	0.009	0.842	0.069	0.552	-0.086	0.129
Weight, kg	0.024	0.402	0.058	0.419	-0.039	0.256
BMI, kg/m^2	0.091	0.429	0.152	0.597	-0.050	0.722
WHR	-11.382	0.026	-22.119	0.079	1.846	0.768
Waist, cm	-0.022	0.559	-0.131	0.167	-0.013	0.781
Hip, cm	0.066	0.182	-0.017	0.891	-0.039	0.514
Thigh, cm	0.141	0.050	0.318	0.074	-0.179	0.038
Fat Mass Index, kg/m^2	0.082	0.587	-0.551	0.144	0.089	0.624
Lean Mass Index, kg/m^2	0.170	0.431	1.577	0.003	-0.363	0.163
Visceral mass index, kg/m^2	-0.347	0.803	-6.717	0.053	0.123	0.941
Android fat mass, kg	0.000	0.677	-0.001	0.169	-1.914	0.962
Gynoid fat mass, kg	0.000	0.287	-0.001	0.410	4.275	0.915
Gynoid lean mass, kg	0.000	0.247	0.002	0.007	-0.001	0.010
Legs fat mass, kg	0.000	0.308	-0.001	0.198	7.311	0.739
Legs lean mass, kg	6.847	0.569	0.001	0.071	0.000	0.110
AF/GF	0.136	0.936	-2.186	0.605	-1.450	0.485
GF/TF	1.311	0.748	-22.241	0.027	-0.219	0.964
AF/TF	1.208	0.793	-26.101	0.023	-1.840	0.738
LF/TF	0.698	0.755	-14.404	0.009	0.401	0.881
GL/TL	1.076	0.872	39.080	0.016	-17.859	0.026
LL/TL	-0.291	0.918	13.539	0.050	-6.063	0.073

AF/GF, android fat/gynoid fat; GF/TF, gynoid fat/total fat; AF/TF, android fat/total fat; LF/TF legs fat/total fat; GL/TL gynoid lean/total lean; AL/TL android lean/total lean; LL/TL legs lean/total lean

Model 1: adjusted for age.

Table S5. Multivariable linear regression analysis adjusted by age, history of CVD (i.e. arterial hypertension (AH), atrial fibrillation (AF), MI, coronary heart disease (CHD), peripheral artery disease (PAD), stroke), history of diabetes mellitus (Model 2) between body composition and subjective well-being scores in non-obese women.

Variable	SWLS		EQ-VAS		BDI	
	B	*p*	B	*p*	B	*p*
Height, cm	0.012	0.810	0.115	0.348	-0.074	0.217
Weight, kg	0.013	0.691	-0.079	0.322	-0.043	0.267
BMI, kg/m^2	0.020	0.841	-0.434	0.071	-0.059	0.619
WHR	-14.458	0.001	-25.719	0.018	10.203	0.054
Waist, cm	-0.060	0.119	-0.257	0.006	0.018	0.692
Hip, cm	0.051	0.181	-0.082	0.376	-0.065	0.149
Thigh, cm	0.086	0.137	-0.027	0.848	-0.104	0.133
Fat Mass Index, kg/m^2	-0.007	0.957	-0.756	0.023	-0.068	0.674
Lean Mass Index, kg/m^2	0.184	0.433	-0.236	0.681	-0.142	0.618
Visceral mass index, kg/m^2	-3.706	0.078	-19.218	<0.001	3.492	0.156
Android fat mass, kg	0.000	0.608	-0.002	0.014	9.709	0.844
Gynoid fat mass, kg	0.000	0.474	-0.001	0.168	0.000	0.269
Gynoid lean mass, kg	0.001	0.094	0.001	0.560	-0.001	0.077
Legs fat mass, kg	0.000	0.308	-0.001	0.198	7.311	0.739
Legs lean mass, kg	6.847	0.569	0.001	0.071	0.000	0.110
AF/GF	-3.729	0.106	-13.004	0.019	3.202	0.239
GF/TF	-7.160	0.075	-3.709	0.698	4.471	0.368
AF/TF	-13.046	0.084	-22.513	0.217	10.832	0.235
LF/TF	-2.881	0.151	0.317	0.947	1.556	0.533
GL/TL	7.042	0.447	21.530	0.337	-26.193	0.018
LL/TL	1.992	0.599	10.283	0.263	-10.571	0.019

AF/GF, android fat/gynoid fat; GF/TF, gynoid fat/total fat; AF/TF, android fat/total fat; LF/TF legs fat/total fat; GL/TL gynoid lean/total lean; AL/TL android lean/total lean; LL/TL legs lean/total lean

Model 2: adjusted for age, history of cardiovascular diseases (i.e. arterial hypertension, atrial fibrillation, myocardial infarction, coronary heart disease, peripheral artery disease, stroke) and history of diabetes mellitus.

Table S6. Multivariable linear regression analysis adjusted by age, history of CVD (i.e. arterial hypertension (AH), atrial fibrillation (AF), MI, coronary heart disease (CHD), peripheral artery disease (PAD), stroke), history of diabetes mellitus (Model 2) between body composition and subjective well-being scores in non-obese men.

Variable	SWLS		EQ-VAS		BDI	
	B	*p*	B	*p*	B	*p*
Height, cm	-0.007	0.891	0.005	0.966	-0.081	0.164
Weight, kg	0.018	0.531	0.061	0.399	-0.0437	0.221
BMI, kg/m^2	0.089	0.460	0.278	0.347	-0.081	0.581
WHR	-11.195	0.039	-11.232	0.391	0.756	0.911
Waist, cm	-0.020	0.614	-0.067	0.498	-0.022	0.654
Hip, cm	0.067	0.197	-0.002	0.984	-0.044	0.481
Thigh, cm	0.143	0.055	0.359	0.046	-0.178	0.047
Fat Mass Index, kg/m^2	0.067	0.671	-0.361	0.350	0.071	0.708
Lean Mass Index, kg/m^2	0.189	0.395	1.564	0.003	-0.413	0.128
Visceral mass index, kg/m^2	-0.455	0.758	-4.885	0.177	-0.151	0.933
Android fat mass, kg	0.000	0.738	-0.001	0.433	-6.105	0.885
Gynoid fat mass, kg	0.000	0.413	-0.001	0.501	2.307	0.956
Gynoid lean mass, kg	0.000	0.325	0.002	0.010	-0.001	0.011
Legs fat mass, kg	0.000	0.462	-0.001	0.191	7.489	0.743
Legs lean mass, kg	4.781	0.700	0.000	0.104	0.000	0.115
AF/GF	0.343	0.848	1.673	0.697	-1.728	0.430
GF/TF	1.195	0.777	-19.438	0.055	-0.772	0.880
AF/TF	1.810	0.708	-19.796	0.093	-2.836	0.627
LF/TF	0.557	0.809	-13.508	0.015	0.222	0.937
GL/TL	0.123	0.986	36.455	0.025	-19.834	0.017
LL/TL	-0.708	0.808	12.470	0.071	-6.789	0.053

AF/GF, android fat/gynoid fat; GF/TF, gynoid fat/total fat; AF/TF, android fat/total fat; LF/TF legs fat/total fat; GL/TL gynoid lean/total lean; AL/TL android lean/total lean; LL/TL legs lean/total lean

Model 2: adjusted for age, history of cardiovascular diseases (i.e. arterial hypertension, atrial fibrillation, myocardial infarction, coronary heart disease, peripheral artery disease, stroke) and history of diabetes mellitus.

Table S7. Multivariable linear regression analysis adjusted by age and WHR (Model 3) between body composition and subjective well-being scores in non-obese women.

Variable	SWLS		EQ-VAS		BDI	
	B	*p*	B	*p*	B	*p*
Height, cm	-0.007	0.893	0.099	0.418	-0.062	0.297
Weight, kg	0.025	0.436	-0.068	0.398	-0.049	0.209
BMI, kg/m^2	0.085	0.385	-0.388	0.116	-0.093	0.433
Waist, cm	0.023	0.629	-0.224	0.058	-0.054	0.340
Hip, cm	0.021	0.578	-0.164	0.086	-0.045	0.324
Thigh, cm	0.065	0.247	-0.060	0.670	-0.074	0.279
Fat Mass Index, kg/m^2	0.069	0.604	-0.745	0.027	-0.109	0.497
Lean Mass Index, kg/m^2	0.319	0.175	0.086	0.884	-0.205	0.476
Visceral mass index, kg/m^2	-0.983	0.640	-20.103	<0.001	1.433	0.569
Android fat mass, kg	0.000	0.694	-0.002	0.026	0.000	0.692
Gynoid fat mass, kg	0.000	0.642	-0.001	0.088	0.000	0.465
Gynoid lean mass, kg	0.001	0.145	0.001	0.539	-0.001	0.117
Legs fat mass, kg	0.000	0.311	0.000	0.382	0.000	0.200
Legs lean mass, kg	0.000	0.270	0.000	0.369	0.000	0.095
AF/GF	-0.119	0.961	-10.735	0.075	-0.361	0.901
GF/TF	-8.057	0.039	-9.114	0.343	6.610	0.178
AF/TF	-8.849	0.238	-25.721	0.167	7.942	0.388
LF/TF	-3.582	0.067	-2.631	0.585	2.787	0.259
GL/TL	8.821	0.332	22.665	0.318	-26.692	0.016
LL/TL	3.128	0.402	11.583	0.215	-11.201	0.013

AF/GF, android fat/gynoid fat; GF/TF, gynoid fat/total fat; AF/TF, android fat/total fat; LF/TF legs fat/total fat; GL/TL gynoid lean/total lean; AL/TL android lean/total lean; LL/TL legs lean/total lean
Model 3: adjusted for age and WHR.

Table S8. Multivariable linear regression analysis adjusted by age and WHR (Model 3) between body composition and subjective well-being scores in non-obese men.

Variable	SWLS		EQ-VAS		BDI	
	B	*p*	B	*p*	B	*p*
Height, cm	0.011	0.814	0.068	0.554	-0.086	0.133
Weight, kg	0.059	0.054	0.123	0.111	-0.050	0.181
BMI, kg/m^2	0.301	0.023	0.521	0.113	-0.090	0.578
Waist, cm	0.071	0.176	-0.031	0.814	-0.042	0.512
Hip, cm	0.065	0.186	-0.023	0.851	-0.038	0.520
Thigh, cm	0.185	0.011	0.393	0.030	-0.192	0.029
Fat Mass Index, kg/m^2	0.349	0.046	-0.289	0.510	0.084	0.689
Lean Mass Index, kg/m^2	0.264	0.230	1.737	0.001	-0.376	0.156
Visceral mass index, kg/m^2	2.543	0.147	-4.564	0.296	-0.320	0.880
Android fat mass, kg	0.001	0.037	0.000	0.647	0.000	0.805
Gynoid fat mass, kg	0.001	0.053	0.000	0.779	1.049	0.980
Gynoid lean mass, kg	0.000	0.166	0.002	0.005	-0.001	0.011
Legs fat mass, kg	0.000	0.093	0.000	0.382	5.894	0.796
Legs lean mass, kg	0.000	0.371	0.001	0.049	0.000	0.107
AF/GF	3.835	0.072	3.635	0.492	-2.732	0.289
GF/TF	4.173	0.322	-18.772	0.069	-0.786	0.878
AF/TF	7.320	0.153	-20.935	0.098	-3.296	0.594
LF/TF	1.943	0.395	-12.841	0.021	0.144	0.958
GL/TL	4.347	0.529	45.772	0.006	-18.694	0.025
LL/TL	1.219	0.676	16.716	0.018	-6.447	0.067

AF/GF, android fat/gynoid fat; GF/TF, gynoid fat/total fat; AF/TF, android fat/total fat; LF/TF legs fat/total fat; GL/TL gynoid lean/total lean; AL/TL android lean/total lean; LL/TL legs lean/total lean
Model 3: adjusted for age and WHR.

Data from the analysis of the population after exclusion of patients with a history of cardiovascular disease (atrial hypertension, atrial fibrillation, myocardial infarction, coronary heart disease, peripheral artery disease, stroke), heart failure, diabetes mellitus, chronic obstructive pulmonary disease, asthma, cancer, chronic kidney disease with glomerular filtration rate <60ml/min, and any mental disorder).

Table S9. Characteristics of the sub population without significant diseases.

**Variable**	**Total population *n* = 411**	**Woman *n* = 227**	**Man *n* = 184**	***p***
Age, years	41.50±13.83	42.12±14.08	40.75±13.52	0.265
SWLS	23.26±5.34	23.12±5.29	23.31±5.42	0.79
EQ-VAS	80.46±13.11	78.46±13.53	82.95±12.14	<0.001
BDI	5.94±5.90	6.67±6.20	5.04±5.39	<0.006
Height, cm	171.86±9.51	165.82±6.53	179.30±7.02	<0.001
Weight, kg	71.13±12.86	63.62±8.66	80.40±11.01	<0.001
Waist, cm	80.47±10.31	74.89±8.05	87.47±8.39	<0.001
Hip, cm	95.29±6.91	95.15±7.41	95.46±6.23	0.522
Thigh, cm	56.41±4.78	56.02±4.75	56.90±4.80	0.040
BMI, kg/m^2	23.97±3.11	23.16±3.12	24.97±2.79	<0.001
BMI <25 kg/m^2	243(59.1)	159 (70)	84 (45)	<0.001
BMI 25-29.99 kg/m^2	168 (40.9)	68 (30)	100 (54.3)	<0.001
WHR	0.84±0.09	0.79±0.06	0.92±0.07	<0.001
WHR, ≥0.85 women, ≥0.9 men	147 (35.8)	35 (15.4)	112 (60.9)	<0.001
Lean Mass Index, (kg/m2)	16.06±2.08	14.63±1.20	17.79±1.54	<0.001
Fat Mass Index, (kg/m^2)	7.18±2.30	7.82±2.29	6.40±2.05	<0.001
Android fat mass, kg	1.73±0.84	1.55±0.73	1.96±0.91	<0.001
Gynoid fat mass, kg	3.47±1.05	3.88±1.00	2.97±0.89	<0.001
Gynoid lean mass, kg	7.16±1.56	6.00±0.65	8.56±1.12	<0.001
Legs fat mass, kg	6.69±2.14	7.70±1.97	5.45±1.64	<0.001
Legs lean mass, kg	16.46±3.76	13.80±1.82	19.69±2.86	<0.001
AF/GF	0.50±0.21	0.38±0.14	0.64±0.20	<0.001
GF/TF	0.15±0.07	0.16±0.07	0.14±0.07	<0.001
AF/TF	0.08±0.05	0.06±0.04	0.09±0.06	<0.001
LF/TF	0.29±0.14	0.32±0.14	0.26±0.14	<0.001
GL/TL	0.15±0.05	0.12±0.03	0.18±0.05	<0.001
LL/TL	0.35±0.12	0.29±0.08	0.42±0.12	<0.001

The data is shown as n (%), mean ± SD. SD, standard deviation; SWLS, Satisfaction With Life Scale; EQ-VAS, Euro Quality of Life Visual Analogue Scale; BDI, Beck Depression Inventory; BMI, body mass index; kg, kilogram; m2, square meter; WHR, waist-hip ratio; AF/GF, android fat/gynoid fat; GF/TF, gynoid fat/total fat; AF/TF, android fat/total fat; LF/TF legs fat/total fat; GL/TL, gynoid lean/total lean; LL/TL, legs lean/total lean.

Table S10. Univariate linear regression analysis between body composition and subjective well-being scores in non-obese women without significant diseases.

Variable	SWLS		EQ-VAS		BDI	
	B	*p*	B	*p*	B	*p*
Age, years	-0.042	0.114	-0.217	0.001	0.025	0.402
Height, cm	0.072	0.190	0.376	0.007	-0.095	0.142
Weight, kg	-0.015	0.718	-0.129	0.221	-0.048	0.334
BMI, kg/m^2	-0.152	0.199	-0.864	0.003	0.001	0.994
WHR	-20.674	<0.001	-33.583	0.016	13.017	0.040
Waist, cm	-0.113	0.014	-0.352	0.002	0.051	0.343
Hip, cm	0.022	0.659	-0.194	0.116	-0.052	0.372
Thigh, cm	0.100	0.200	0.058	0.762	-0.127	0.162
Fat Mass Index, kg/m^2	-0.261	0.107	-1.102	0.006	0.022	0.907
Lean Mass Index, kg/m^2	0.080	0.795	-1.893	0.014	-0.103	0.778
Visceral mass index, kg/m^2	-8.328	0.002	-25.551	<0.001	3.150	0.297
Android fat mass, kg	-0.001	0.049	-0.004	0.005	0.001	0.732
Gynoid fat mass, kg	5.851	0.877	-0.001	0.303	0.000	0.403
Gynoid lean mass, kg	0.001	0.035	0.002	0.277	-0.001	0.025
Legs fat mass, kg	0.000	0.430	1.501	0.975	0.000	0.157
Legs lean mass, kg	0.000	0.170	0.000	0.505	0.000	0.051
AF/GF	-9.262	0.001	-22.566	0.001	4.024	0.216
GF/TF	-9.193	0.073	-7.544	0.559	7.244	0.233
AF/TF	-27.276	0.004	-52.683	0.031	21.299	0.060
LF/TF	-3.941	0.125	0.075	0.991	2.891	0.343
GL/TL	0.472	0.967	11.676	0.681	-20.785	0.116
LL/TL	-0.673	0.885	4.857	0.676	-8.454	0.122

AF/GF, android fat/gynoid fat; GF/TF, gynoid fat/total fat; AF/TF, android fat/total fat; LF/TF legs fat/total fat; GL/TL gynoid lean/total lean; LL/TL legs lean/total lean.

Table S11 Univariate linear regression analysis between body composition and subjective well-being scores in non-obese men without significant diseases

Variable	SWLS		EQ-VAS		BDI	
	B	*p*	B	*p*	B	*p*
Age, years	0.016	0.602	-0.245	<0.001	0.018	0.558
Height, cm	0.044	0.447	0.233	0.073	-0.117	0.046
Weight, kg	0.071	0.056	0.189	0.025	-0.083	0.026
BMI, kg/m^2	0.268	0.073	0.474	0.154	-0.103	0.183
WHR	-7.785	0.228	-30.170	0.032	2.380	0.713
Waist, cm	0.047	0.346	-0.092	0.409	-0.053	0.290
Hip, cm	0.176	0.008	0.163	0.265	-0.130	0.053
Thigh, cm	0.212	0.016	0.788	<0.001	-0.259	0.003
Fat Mass Index, kg/m^2	0.190	0.344	-0.204	0.648	-0.046	0.819
Lean Mass Index, kg/m^2	0.537	0.052	1.881	0.002	-0.605	0.028
Visceral mass index, kg/m^2	1.112	0.544	-7.466	0.062	0.041	0.982
Android fat mass, kg	0.000	0.267	0.000	0.724	0.000	0.619
Gynoid fat mass, kg	0.001	0.192	0.001	0.521	0.000	0.501
Gynoid lean mass, kg	0.001	0.020	0.003	<0.001	-0.001	0.001
Legs fat mass, kg	0.000	0.453	6.990	0.901	0.000	0.536
Legs lean mass, kg	0.000	0.124	0.001	0.001	0.000<br>**β** -0.188	0.015
AF/GF	1.982	0.345	-3.330	0.464	-0.763	0.719
GF/TF	6.158	0.268	0.536	0.965	-1.677	0.763
AF/TF	10.561	0.127	-0.131	0.993	-3.301	0.633
LF/TF	2.162	0.479	-2.263	0.735	-0.644	0.833
GL/TL	12.109	0.158	41.732	0.023	-17.441	0.041
LL/TL	4.022	0.267	17.054	0.028	-6.393	0.077

AF/GF, android fat/gynoid fat; GF/TF, gynoid fat/total fat; AF/TF, android fat/total fat; LF/TF legs fat/total fat; GL/TL gynoid lean/total lean; LL/TL legs lean/total lean.

Table S12. Multivariable linear regression analysis adjusted by age (Model 1) between body composition and subjective well-being scores in non-obese women without significant diseases.

Variable	SWLS		EQ-VAS		BDI	
	B	*p*	B	*p*	B	*p*
Height, cm	0.044	0.472	0.223	0.140	-0.088	0.221
Weight, kg	-0.004	0.926	-0.056	0.595	-0.058	0.256
BMI, kg/m^2	-0.087	0.505	-0.525	0.106	-0.064	0.680
WHR	-20.091	0.001	-18.706	0.207	13.242	0.062
Waist, cm	-0.101	0.051	-0.216	0.096	0.038	0.536
Hip, cm	0.045	0.386	-0.085	0.502	-0.070	0.249
Thigh, cm	0.101	0.194	0.091	0.631	-0.128	0.157
Fat Mass Index, kg/m^2	-0.201	0.255	-0.637	0.150	-0.050	0.812
Lean Mass Index, kg/m^2	0.272	0.409	-1.097	0.178	-0.243	0.537
Visceral mass index, kg/m^2	-8.833	0.005	-20.086	0.008	2.759	0.445
Android fat mass, kg	-0.001	0.120	-0.002	0.104	3.199	0.961
Gynoid fat mass, kg	0.000	0.763	-0.001	0.553	0.000	0.353
Gynoid lean mass, kg	0.001	0.052	0.001	0.442	-0.001	0.031
Legs fat mass, kg	0.000	0.370	0.000	0.653	0.000	0.135
Legs lean mass, kg	0.000	0.227	0.000	0.707	0.000	0.062
AF/GF	-0.231	0.003	-15.388	0.042	3.575	0.329
GF/TF	-9.168	0.073	-8.356	0.508	7.294	0.231
AF/TF	-25.418	0.010	-33.723	0.174	20.314	0.084
LF/TF	-3.955	0.123	-0.371	0.953	2.925	0.337
GL/TL	2.231	0.843	19.994	0.472	-22.032	0.097
LL/TL	-0.075	0.987	7.770	0.495	-8.869	0.106

AF/GF, android fat/gynoid fat; GF/TF, gynoid fat/total fat; AF/TF, android fat/total fat; LF/TF legs fat/total fat; GL/TL gynoid lean/total lean; LL/TL legs lean/total lean
Model 1: adjusted for age.

Table S13. Multivariable linear regression analysis adjusted by age (Model 1) between body composition and subjective well-being scores in non-obese men without significant diseases.

Variable	SWLS		EQ-5D		BDI	
	B	*p*	B	*p*	B	*p*
Height, cm	0.062	0.319	0.072	0.593	-0.120	0.057
Weight, kg	0.073	0.051	0.163	0.046	-0.082	0.029
BMI, kg/m^2	0.262	0.084	0.675	0.037	-0.220	0.150
WHR	-10.821	0.123	-11.364	0.447	1.220	0.863
Waist, cm	0.043	0.420	0.047	0.677	-0.069	0.197
Hip, cm	0.175	0.009	0.189	0.180	-0.132	0.051
Thigh, cm	0.236	0.009	0.650	0.001	-0.263	0.003
Fat Mass Index, kg/m^2	0.177	0.388	0.149	0.735	-0.071	0.728
Lean Mass Index, kg/m^2	0.534	0.054	1.850	0.001	-0.608	0.028
Visceral mass index, kg/m^2	0.854	0.680	-0.341	0.939	-0.584	0.778
Android fat mass, kg	0.000	0.309	0.001	0.571	0.000	0.523
Gynoid fat mass, kg	0.001	0.183	0.000	0.652	0.000	0.521
Gynoid lean mass, kg	0.001	0.012	0.002	0.005	-0.001	0.002
Legs fat mass, kg	0.000	0.433	-6.374	0.906	0.000	0.560
Legs lean mass, kg	0.000	0.084	0.001	0.015	0.000<br>β -0.190	0.018
AF/GF	1.848	0.426	4.944	0.312	-1.572	0.502
GF/TF	6.589	0.240	-5.418	0.648	-1.296	0.817
AF/TF	10.383	0.135	3.304	0.825	-3.585	0.606
LF/TF	2.387	0.439	-5.683	0.383	-0.426	0.890
GL/TL	12.430	0.149	35.682	0.045	-17.166	0.046
LL/TL	4.202	0.249	14.099	0.063	-6.249	0.085

AF/GF, android fat/gynoid fat; GF/TF, gynoid fat/total fat; AF/TF, android fat/total fat; LF/TF legs fat/total fat; GL/TL gynoid lean/total lean; LL/TL legs lean/total lean
Model 1: adjusted for age.
